# The Basis of Compensation for Medical Officers

**Published:** 1917-06

**Authors:** 


### The Basis of Compensation for Medical Officers.

Whether in regard to Health Insurance or any similar scheme, it occurs to us that the most practical plan for the medical profession is to insist on a reasonable compensation, in accordance with formally established precedent. The three principal government services afford such a precedent, tested and approved by time. Broadly stated they are that a competent physician is worth from $2000 a year at the start to $4200 after 20 years of experience, with possibility of further promotion for longer or unusually valuable service. In addition, reasonable vacations, sick leave and pensions on retirement and quarters or allowance therefor at $30-$50 a month are provided. These salaries and perquisites are net, equipments of all kinds for professional services being provided. When we say that this basis of compensation has been tested and approved by time, we mean that experience has shown that it is just about what is required to comply with the balance between supply and demand of competent service. There is no reason why a state, county, city or other conceivable branch of government should, on the one hand, expect to secure competent service at a less rate and, on the other hand, why a mistaken sense of professional dignity should overlook actual facts and attempt to secure a higher rating.

